# Novel Protein kinase C θ: Coronin 1A complex in T lymphocytes

**DOI:** 10.1186/s12964-015-0100-3

**Published:** 2015-03-31

**Authors:** Kerstin Siegmund, Nikolaus Thuille, Nina Posch, Friedrich Fresser, Gottfried Baier

**Affiliations:** Department for Pharmacology and Genetics, Division of Translational Cell Genetics, Medical University Innsbruck, Peter Mayr Str. 1a, A-6020 Innsbruck, Austria

**Keywords:** T lymphocyte signaling, Transcriptional regulation, Protein kinase C θ (PKCθ), Coronin 1A (Coro1A), NF- κB, IL-2

## Abstract

**Background:**

Protein kinase C-θ (PKCθ) plays an important role in signal transduction down-stream of the T cell receptor and T cells deficient of *PKCθ* show impaired NF-κB as well as NFAT/AP-1 activation resulting in strongly decreased IL-2 expression and proliferation. However, it is not yet entirely clear, how the function of PKCθ - upon T cell activation - is regulated on a molecular level.

**Findings:**

Employing a yeast two-hybrid screen and co-immunoprecipitation analyses, we here identify coronin 1A (Coro1A) as a novel PKCθ-interacting protein. We show that the NH_2_-terminal WD40 domains of Coro1A and the C2-like domain of PKCθ are sufficient for the interaction. Furthermore, we confirm a physical interaction by GST-Coro1A mediated pull-down of endogenous PKCθ protein. Functionally, wild-type but not Coro1A lacking its actin-binding domain negatively interferes with PKCθ-dependent NF-κB, Cyclin D1 and IL-2 transactivation when analysed with luciferase promoter activation assays in Jurkat T cells. This could be phenocopied by pharmacological inhibitors of actin polymerization and PKC, respectively. Mechanistically, Coro1A overexpression attenuates both lipid raft and plasma membrane recruitment of PKCθ in CD3/CD28-activated T cells.

Using primary CD3^+^ T cells, we observed that (opposite to PKCθ) Coro1A does not localize preferentially to the immunological synapse. In addition, we show that CD3^+^ T cells isolated from *Coro1A*-deficient mice show impaired IKK/NF-κB transactivation.

**Conclusions:**

Together, these findings both in Jurkat T cells as well as in primary T cells indicate a regulatory role of Coro1A on PKCθ recruitment and function downstream of the TCR leading to NF-κB transactivation.

**Electronic supplementary material:**

The online version of this article (doi:10.1186/s12964-015-0100-3) contains supplementary material, which is available to authorized users.

## Findings

An efficient adaptive immune response depends on the activation of T lymphocytes by antigen-presenting cells (APC) and the acquisition of appropriate effector T cell function. Activation of T lymphocytes occurs upon engagement of T cell receptors (TCR) and the corresponding antigen-MHC complexes together with the ligation of the co-receptor CD28 by B7 molecules – leading to the formation of the immunological synapse (IS) between T cell and APC. PKCθ, a member of the protein kinase C (PKC) family of serine/threonine kinases, is rapidly, within seconds after TCR engagement, recruited to the peripheral supra-molecular activation cluster (pSMAC) of the immunological synapse (IS) [1,2]. It has been shown that, the activation of the transcription factors NF-κB, NFAT and AP-1 downstream of the TCR critically depend on PKCθ [3-5], linking PKCθ function to IL-2 transcription, whose promoter activation depends on these transcription factors [6].

Activation of all PKC family members is controlled by a so-called pseudo-substrate (PS) domain in the NH_2_-terminus that resembles PKC substrates and forms an auto-inhibitory loop to keep the enzyme in an inactive conformation. PKCθ is released from the auto-inhibition after recruitment to the plasma membrane, where it binds to diacylglycerol (reviewed in [7]). Noteworthy, PKC mutations that disrupt this intra-molecular interaction generate constitutively active forms of PKC, which are useful tools for analysing PKC functions. Another domain involved in modulating PKCθ activity is its C2-like domain, which represents a major protein:protein interaction domain. Binding of WD40 domain-containing receptor for activated PKC proteins – so called RACK proteins - to the C2-like domain of activated PKC forms another level of regulating its enzymatic function [8]. However, so far no RACK physiologically interacting with PKCθ has been identified. In the present study, designed to discover PKCθ interacting partners, we identified coronin 1A (Coro1A) as a functional regulator of PKCθ activation.

## Identification of Coro1A as physical interaction partner of PKCθ

Little is known about proteins that interact with PKCθ and regulate its function in T lymphocytes and thereby modulate activation of this immune cell subset. To contribute to this issue, we have employed a yeast two-hybrid (Y2H) screen using the regulatory domain of PKCθ (PKCθ-NH_2_) fused to the DNA-binding domain as “bait”. With this approach we identify a group of clones that interact strongly with PKCθ. A detailed description of all methods used is provided in the Additional file 1 (Supplementary Methods). DNA sequencing reveals the interacting “prey” protein as the NH_2_-terminal domain of human Coro1A (Table 1). Coro1A, a member of the evolutionary conserved WD-repeat family of coronin proteins, is highly expressed in all leukocytes. Originally, Coro1A has been isolated as an actin/myosin binding protein and implicated in F-actin dynamics by negatively regulating the function of the nucleation-promoting Arp2/3 complex (reviewed in [9]). In mice and human, genetic inactivation of Coro1A results in immune deficiencies that are linked to a strong reduction of naive T cell numbers in peripheral organs [10-15]. Of note, Coro1A has been implicated in calcium mobilization after TCR triggering in naive T cells as well as TGF-β signaling in Th17 cells [11,14].

**Table 1:** **Specific interaction between PKCθ and Coronin 1A in the GAL4 two-hybrid system**

**DNA-binding domain hybrid “bait”**	**Activation-domain hybrid “prey”s**	**Leu-protothrophy**
PKCθ NH2-terminus	control	**-**
PKCθ COOH-terminus	control	**-**
PKCθ C2-like domain	control	**-**
PKCα NH2-terminus	control	**-**
*PKC* *θ* *NH2-terminus*	*Coronin 1A wt*	**+**
PKCθ COOH-terminus	Coronin 1A wt	**-**
PKCα NH2-terminus	Coronin 1A wt	**-**
*PKC* *θ* *C2-like domain*	*Coronin 1A wt*	**++**
*PKC* *θ* *NH2-terminus*	*Coronin 1A mutant*	**+**
PKCθ COOH-terminus	Coronin 1A mutant	**-**
PKCα NH2-terminus	Coronin 1A mutant	**-**
*PKCθ C2-like domain*	*Coronin 1A mutant*	**++**
control	Coronin 1A wt	**-**
control	Coronin 1A wt	**-**
control	Coronin 1A mutant	**-**
control	Coronin 1A mutant	**-**

PKCθ and Coro1A were identified as interaction partners applying the GAL4 Two-Hybrid System. Saccharomyces cerevisiae reporter strain EGY48 was co-transformed with the bait construct encoding the NH_2_-terminal regulatory domain of PKCθ fused to a GAL4 DNA-binding domain and the human lymphocyte Matchmaker cDNA library. In re-transformation analysis, the NH_2_ and COOH domain of PKCθ were tested for interaction with truncated versions of Coro1A as indicated.

**Figure 1:**
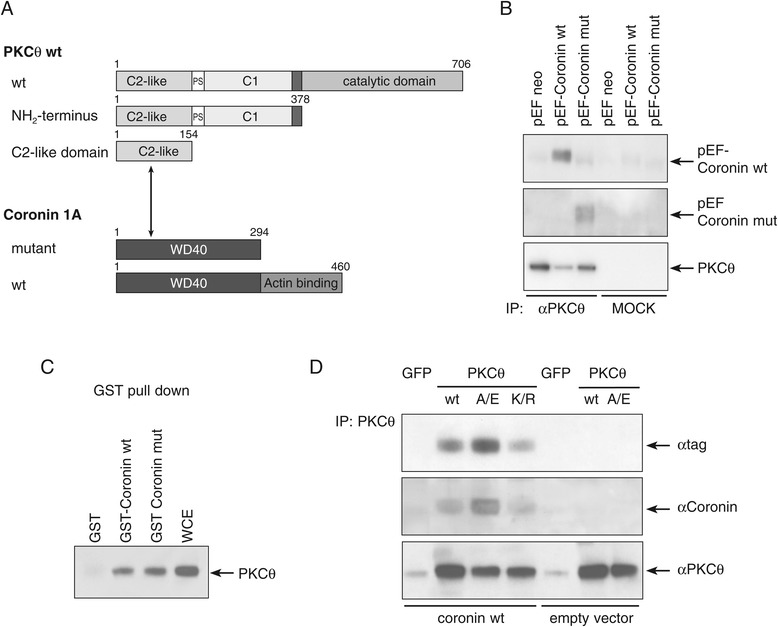
**PKCθ physically interacts with Coro1A. (A)** The cartoon depicts interactions identified between deletion mutants of PKCθ and Coro1A by Y2H as well as Co-IP experiments. It could be shown by deletion assays that the WD40 domain of Coro1A and the C2-like domain of PKCθ are sufficient for their interaction. **(B)** Co-immunoprecipitation analysis of Jurkat T cells transiently transfected with empty vector pEF-neo or RGSH_6_-tagged Coro1A constructs (wild-type or deletion mutant). Twenty-four hours after transfection, PKCθ-specific immunoprecipitates were immunostained with antibodies recognizing the tag, PKCθ or Coro1A. To control for the specificity of the interaction immunoprecipitation with control IgG (MOCK) was performed. **(C)** GST-Coro1A pull-down of endogenous PKCθ from mouse CD3^+^ T cells. WCE = whole cell extract as positive control was used in parallel. **(D)** Jurkat T cells were transiently transfected with empty vector or RGSH_6_-tagged wild-type Coro1A together with GFP or PKCθ allelic versions (wild-type, A/E – constitutively active and K/R – dominant negative). Twenty-four hours after transfection, PKCθ-specific antibodies were used for immunoprecipitation and the pulled-down proteins were analysed by immunoblotting the fusion-tag as well as Coro1A and PKCθ-specific antibodies. Experiments were repeated at least three times, with similar results.

Using truncated versions of PKCθ and Coro1A (Figure 1A), we demonstrate that the N-terminal WD40 domains of Coro1A and the C2-like domain of PKCθ are sufficient for the interaction. Co-immunoprecipitation (Co-IP) analysis in Jurkat T cells transfected with an epitope-tagged Coro1A expression vector confirmed a complex formation between PKCθ and Coro1A in T cells (Figure 1B). Reversely, GST-Coro1A pull-downs revealed interaction with endogenous PKCθ in mouse T cells (Figure 1C). This PKCθ:Coro1A interaction was observed both with and without CD3/CD28 stimulation of the cells and thus being constitutive in resting cells. Of note, the Co-IP experiments show strongly increased physical association of Coro1A with the constitutively active mutant PKCθ A149E, while the binding to the dominant-negative PKCθ K409R mutant remained unaltered when compared to wild-type PKCθ (Figure 1D). This suggests that Coro1A might function as a RACK protein regulating PKC kinase activity. Of note, based on experiments using phorbol ester as pleiotropic PKC activator, or serine/threonine protein phosphatase inhibitors, PKCs have been described as kinases phosphorylating Coro1A and thereby down-regulating its binding to actin [16,17]. Itho *et al.* identified PKCα and PKCδ as the PKC isotypes responsible for Coro1A phosphorylation [18].

## Coro1A modulates PKCθ-mediated functions

**Figure 2:**
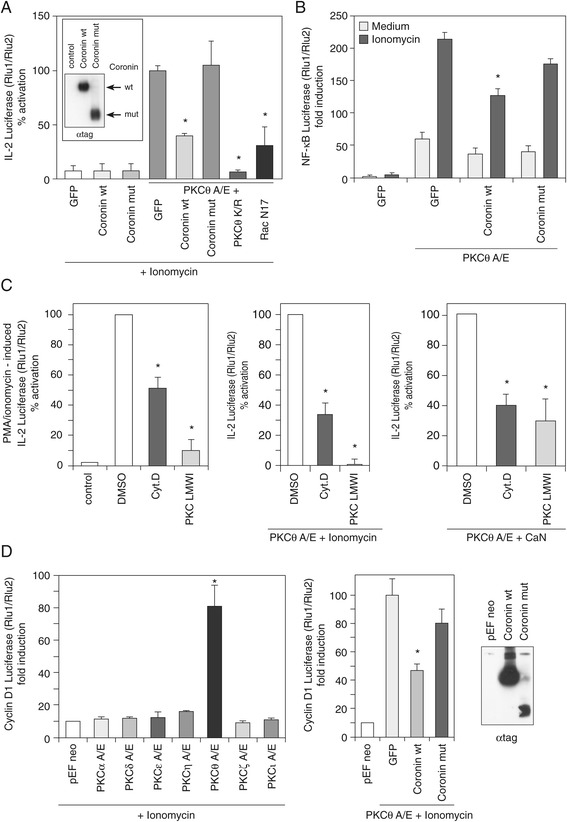
**Coro1A modulates PKCθ-mediated effector function. (A)** IL-2 promoter luciferase reporter assay performed with Jurkat T cells transfected with the constitutively active mutant PKCθ A/E and wild-type or truncated Coro1A – as indicated. Transfected cells were stimulated with the calcium ionophore ionomycin overnight. The insertion in the upper left corner shows expression of recombinant Coro1A in Jurkat T cells analysed by an anti-tag immunoblot. GFP-expressing plasmid was used as an inert protein overexpression control. **(B)** NF-κB-dependent promoter luciferase reporter of transfected Jurkat T cells either stimulated with ionomycin or left untreated. **(C)** IL-2 promoter-dependent luciferase reporter of Jurkat cells stimulated with phorbol ester/ionomycin, transfected with constitutively active mutant PKCθ A/E and stimulated with ionomycin, or alternatively, transfected with constitutively active mutants of both PKCθ and Calcineurin (CaN) and/or treated by cytochalasin (Cyt) D and PKC LMWI AEB071/Sotratstaurin as indicated. **(D)** Cyclin D1 promoter-dependent luciferase reporter of Jurkat cells stimulated with ionomycin and transfected with constitutively active mutants of several PKC family members and with PKCθ A/E and wild-type or truncated Coro1A, respectively. The mean ± SE of three independent experiments is shown. Statistical significance was defined with p < 0.05 (Student’s t-test) and marked with one asterisk (*). A/E: constitutively active and K/R: dominant negative mutant of PKCθ; Rac1 N17: dominant-negative mutant of Rac1; Rlu - relative luciferase activity.

After having observed a complex formation between PKCθ and Coro1A, we next asked the question about the functional relevance of this interaction. Therefore, it was analysed whether Coro1A does influence the transcriptional activation of genes that are established downstream targets of PKCθ such as IL-2 and Cyclin D1. In functional analyses using IL-2 promoter luciferase reporter assays, overexpression of wild-type Coro1A but not the COOH-deletion mutant, lacking the actin-binding domain, negatively interferes with PKCθ-dependent IL-2 transactivation in Jurkat T cells (Figure 2A). Thus, even though the actin-binding function of Coro1A is not necessary for its interaction with PKCθ (Figure 1), it appears to be of relevance for Coro1A modulating PKCθ function. In these experiments, Jurkat T cells co-transfected with the constitutively active mutant PKCθ A149E and wild-type or truncated Coro1A, were stimulated with the calcium ionophore, ionomycin. Co-transfection with the dominant-negative PKCθ K409R mutant or the dominant-negative mutant of Rac1, Rac1 N17, which leads to inhibition of IL-2 reporter transcription via actin polymerization defects served as positive controls. Those findings suggest that actin is part of a functional PKCθ:Coro1A axis identified in the Jurkat T cell line. In addition, wild-type but not the deletion mutant of Coro1A repressed the induction of an NF-κB-dependent promoter luciferase reporter (Figure 2B). This effect could be phenocopied both by cell-permeable pharmacological inhibitors of actin polymerisation and PKC function, respectively (Figure 2C). Similarly, Cyclin D1 promoter reporter activation (that was PKC isotype-selectively dependent on PKCθ function) was attenuated by wild-type Coro1A co-expression (Figure 2D).

**Figure 3:**
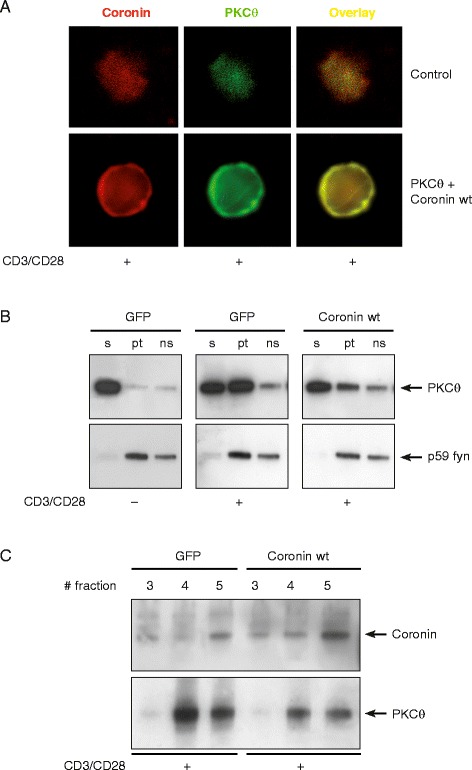
**Coro1A modulates PKCθ-** **mediated subcellular location in activated T cells.** Jurkat cells were transfected with GFP inert protein control, PKCθ or Coro1A wild-type cDNA expression plasmids, respectively. **(A)** Co-localization of transfected PKCθ and Coro1A occurred at a rate of approximately 76% in intact cells as measured by immunofluorescence. Jurkat T cells were stimulated for 20 min with anti-CD3/anti-CD28 antibodies and analysed by subsequent staining with protein-specific antibodies. A representative image is shown. **(B)** Translocation of PKCθ to the plasma membrane is inhibited by overexpression of Coro1A. Jurkat cells were transfected with Coro1A or GFP, respectively. After 21 hrs cells were stimulated with anti-CD3/anti-CD28 antibodies for 20 min or left unstimulated, as indicated and subcellular distribution of endogenous PKCθ was determined by immunoblotting. The cell fractions are defined as the soluble (s) fraction, the particulate (pt) fraction and the Triton-X100 non-soluble (ns) fraction, which were prepared as described in the Additional file 1 (Supplementary Methods). The p59 fyn protein was detected to control for cell fractionation. **(C)** Lipid rafts were prepared by fractionation of sucrose gradients and immunostained for PKCθ and Coro1A. As a marker for the raft fraction, the marker ganglioside M1 of each fraction was quantified in a dot blot employing HRP-Choleratoxin B (not shown). A representative experiment of 3 independent experiments is shown.

Mechanistically, in transient Jurkat transfection assays, PKCθ and Coro1A co-localized in intact Jurkat T cells (Figure 3A), and Coro1A overexpression inhibited both plasma membrane and lipid raft recruitment of PKCθ in CD3/CD28-activated cells (Figure 3B/C). While we cannot exclude additional Coro1A functions affecting NF-κB activation independent of PKCθ, based on the experiments described above, we conclude that Coro1A, which is in a complex with PKCθ, modulates PKCθ functionally.

Taken together, Coro1A likely may act as a safeguard for stochastic membrane recruitment/IS translocation of PKCtheta upon transient T cell activation signals, e.g. by low affinity antigens.

## Coro1A is involved in NF-κB signaling in primary T lymphocytes

**Figure 4:**
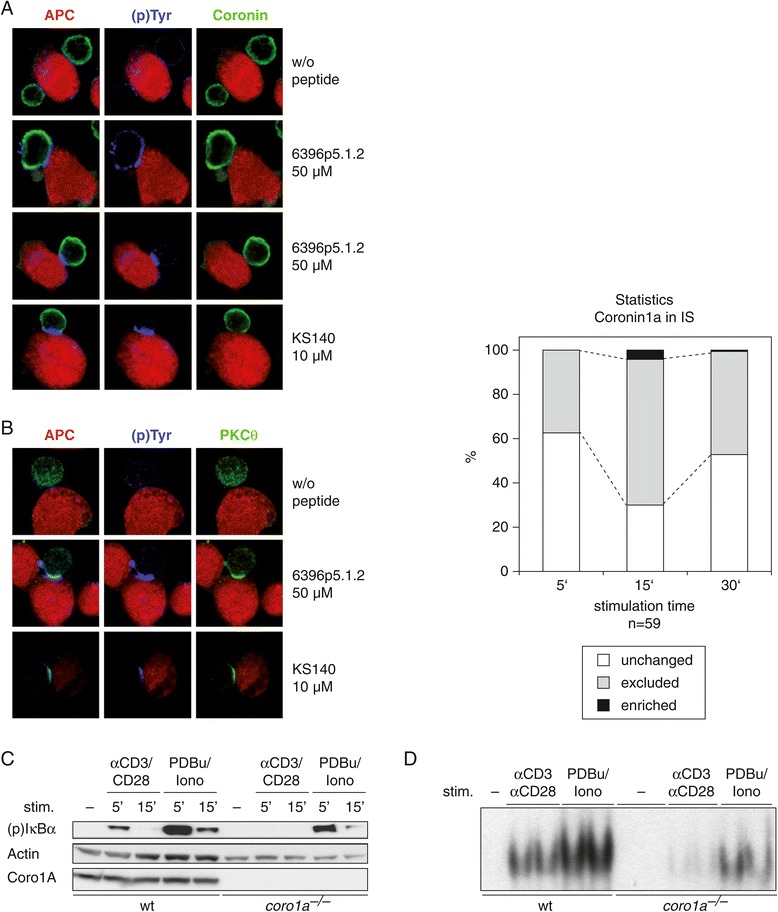
**Coro1A is not recruited into the IS and its gene ablation strongly reduces NF-κB responses. (A, B)** Confocal microscopy of Coro1A and PKCθ in primary human T cells. A T cell clone (KS140) specific for the tetanus toxin peptide (TT830–843; QYIKANSKFIGITE) and a T cell clone (6396p5.1.2) specific for the measles virus fusion protein peptide (F254–268; GDLLGILESRGIKAR) were used with autologous Epstein–Barr virus (EBV)-transformed B cells as APC. Quantification of Coro1A subcellular localization on 59 synapses is shown as bar graph. **(C)** CD3^+^ T cells were isolated from either wild-type or *Coro1a* knockout mice. After 2 hour resting *ex vivo* the cells were stimulated with soluble anti-CD3/CD28 and cross-linking anti-hamster IgG antibodies or PDBu for 5 and 15 minutes. Whole cell lysates (supplemented with phosphatase inhibitor) were subjected to SDS-Page and immunoblotting against phosphorylated IκBα, actin and Coro1A. **(D)** CD3^+^ T cells were isolated and stimulated as described in **(C)**, but the stimulation time was increased to 8 hours. Nuclear extracts were prepared and analysed by electromobility shift assays (EMSA) for NF-κB binding to DNA. Experiments were repeated at least two times, with similar results.

Next, we investigated the subcellular localization of Coro1A and PKCθ upon T cell activation. For this purpose human T cell blasts from immunized donors were incubated with APCs loaded or not with the corresponding peptide and analysed by confocal microscopy for the localization of PKCθ and Coro1A with regard to the IS (stained by antibodies against (p)tyrosine). Of note, while as already published, activation-induced PKCθ recruitment to the IS was consistently observed by confocal microscopy [19], Coro1A was not recruited to the IS. Coro1A was rather excluded from the IS in approximately 65% of antigen:APC-stimulated T cell blasts (Figure 4A/B), suggesting a role as negative regulator in TCR signaling.

Results on the molecular mechanism of Coro1A in T cell signaling are controversial in part due to the diverse results obtained with the different conventional knockout mice strains established in several laboratories [10,11,20]. In particular, Mueller *et al*. described a physical interaction between Coro1A and PLC- γ1 promoting calcium mobilization from intracellular stores upon activation of naive T cells [11], while no defect in other pathways downstream of the TCR was detected. In contrast, Föger *et al*. did not observe any impairment of T cell activation at all when analysing T cell function using their knockout strain [10]. Using our *Coro1a* knockout mice [14], we addressed the potential involvement of Coro1A in the NF-κB signaling pathway, known to be regulated by PKCθ, in primary mouse T cells. The results revealed reduced levels of phosphorylated inhibitor of NF-κB (I-κBα) in T cells isolated from *Coro1a*-deficient mice upon stimulation with anti-CD3 and anti-CD28 (Figure 4C). Furthermore, NF-κB:DNA binding upon anti-CD3/CD28 treatment was strongly reduced in *Coro1a*-deficient T cells when analysed by electrophoretic mobility shift assay (EMSA) (Figure 4D).

Using a combination of phorbol ester and ionomycin, which bypass early activation events downstream of the TCR by directly activating PKC isotypes and inducing calcium influx, respectively, only partially restored I-κBα phosphorylation and NF-κB:DNA binding, pointing to an important role of Coro1A for PKC activation processes.

Taken together, the present results provide evidence that Coro1A is a functional interaction partner of PKCθ in the established PKCθ/IKK/NF-κB/IL-2 transactivation pathway in CD3^+^ T cells.

## Additional file

Additional file 1:
**Supplementary Methods.**

## Abbreviations

APC: Antigen presenting cell
AP-1: Activating protein 1
CaN: Calcineurin
Coro1A: Coronin 1A
Co-IP: Co-immunoprecipitation
CA: Constitutively active
CycD1: Cyclin D1
CytD: Cytochalasin D
DN: Dominant negative
EMSA: Electrophoretic mobility shift assay
GFP: Green fluorescence protein
IL-2: Interleukin-2
IS: Immunological synapse
NFAT: Nuclear factor of activation in T cells
NF-κB: Nuclear factor κ B
PKC: Protein kinase C
PDBu: Phorbol 12,13-dibutyrate
ko: Knockout
PKC LMWI: PKC low molecular weight inhibitor
TCR: T cell receptor
Y2H: Yeast two-hybrid
