# Supplementary material for: Novel Protein kinase C θ: Coronin 1A complex in T lymphocytes
Source: Cell Commun Signal. 2015 Mar 31;13:22. doi: 10.1186/s12964-015-0100-3 (PMC4390099; doi:10.1186/s12964-015-0100-3)
Supplement: Additional file 1: — Supplementary Methods. [file 12964_2015_100_MOESM1_ESM.docx]

# Additional files

**Supplementary methods**

#### Two-hybrid screening and cDNA isolation

#### The Saccharomyces cerevisiae reporter strain EGY48 used for the GAL4 two-hybrid screen was purchased from Clontech Laboratories, Inc. (Palo Alto, CA). S. cerevisiae strains were grown under standard conditions in rich or synthetic medium with appropriate supplements at 30°C. For the yeast two-hybrid screening, EGY48 was co-transformed with the bait construct encoding the regulatory domain of PKCtheta fused to a GAL4 DNA-binding domain and the human lymphocyte Matchmaker cDNA library (Clontech Laboratories, Inc., Palo Alto, CA). Transformants were plated on synthetic dropout medium lacking leucine, tryptophan, and histidine but containing 5 mM 3-amino-1,2,4-triazole. His^+^ colonies were assayed for beta-galactosidase activity using o-nitrophenyl b-D-galactopyranoside as substrate. At least 12 individual co-transformants were assayed.

#### Immunoprecipitation analysis

#### Jurkat TAg cells (1x10^7^) were lysed in 1 ml lysis buffer (50 mM Tris-HCl, pH 7.3, 5 mM NaF, 5 mM Na_3_VO_4_, 2 mM EDTA, 150 mM NaCl, 0.5% TritonX-100, 50 mg/ml aprotinin, 50 mg/ml leupeptin). Lysates were pre-cleared for 30 min at 4°C. Ni^2+^ pull down and immunoprecipitation was performed at 4°C overnight followed by incubation with NTA–agarose (Qiagen) or antibody/protein G Sepharose (Amersham-Pharmacia, Vienna), and immunoprecipitated protein was subjected to SDS-PAGE under reducing conditions on Bis/Tris-buffered gels (Novex, San Diego, CA). Proteins were transferred to a polyvinyldifluoride (PVDF) membrane (Millipore, Bedford, MA) by semi-dry blotting (90 mA/112 cm^2^, 90 min, 4°C). The primary antibodies were diluted in Tris-buffered saline (TBS) containing 0.05% Tween-20 and 5% non-fat dry milk for incubation. Peroxidase-conjugated antibodies (Pierce, Rockford, IL) served as the secondary reagent (1:5000). Enhanced chemiluminescence (Super Signal, Pierce, Rockford, IL) was used for antigen detection.

#### Cell isolation, transfection and stimulation conditions

Jurkat TAg cells were maintained in RPMI medium (Biochrom, Berlin, Germany) supplemented with 10% FCS (Life Technologies, Inc.). Transient transfection of cells was performed by electroporation in a BTX-T820 ElectroSquarePorator (ITC, Biotech, Heidelberg, Germany) apparatus using predetermined optimal conditions (2x10^7^ cells at 450 V/cm, 5 pulses of 99 ms). Murine CD3^+^ T cells were negatively selected and purified from pooled spleen and lymph nodes using mouse T cell enrichment columns according to the manufactures´ instructions (R&D Systems).

#### Reporter Gene Assay

#### For the reporter gene expression assay Jurkat-TAg cells were transfected with the indicated reporter and expression constructs and measured as previously described [[1](#_ENREF_1)].

#### Gel mobility shift assays

Nuclear extracts were harvested from 1×10^7^ cells according to standard protocols. Briefly, purified CD3^+^ T cells were washed in PBS and resuspended in 10 mM HEPES (pH 7.9) 10 mM KCl, 0.1 mM EDTA, 0.1 mM EGTA, 1 mM DTT and protease inhibitors. Cells were incubated on ice for 15 min. NP-40 was added to a final concentration of 0.6%, cells were vortexed vigorously, and the mixture was centrifuged for 5 min. The nuclear pellets were washed twice and resuspended in 20 mM HEPES (pH7.9), 0.4 M NaCl, 1 mM EDTA, 1 mM EGTA, and 1 mM DTT and protease inhibitors, and the tube was rocked for 30 min at 4 °C. After centrifugation for 10 min, the supernatant was collected. Extracted proteins (2 μg) were incubated in binding buffer with [32P]-labeled, double-stranded oligonucleotide probes (NF-kappaB: 5′-GCC ATG GGG GGA TCC CCG AAG TCC-3′) (Nushift; Active Motif). In each reaction, 3 × 10^5^ c.p.m. of labeled probe was used, and the band shifts were resolved on 5% polyacrylamide gels. All experiments were performed at least three times with similar outcomes.

#### Isolation of detergent insoluble membrane (lipid raft) fractions

6x10^7^ Jurkat TAg cells or 3x10^7^ Jurkat E6-1 cells, respectively, per assay point were transfected with 20µg of the cDNA expression plasmids, encoding Coro1a or GFP as inert protein control as indicated. After incubation for 21 hours the cells were stimulated with solid-phase IgG clones of CD3- and/or CD28-specific antibodies for 30 min at 37°C or left unstimulated. Subsequently, cells were lysed in 1ml of ice-cold MES buffer (25mM MES pH6.5, 5mM NaF, 1mM Na_3_VO_4_, 2mM EDTA, 150mM NaCl, 0.5% Triton X-100, 50µg/ml Aprotinin and Leupeptin), passing the cells 30x through a 25G needle. Lysates were mixed with an equal volume of 80% sucrose in MES buffer and overlayed with 6ml of 30% sucrose/MES and finally 3.5ml of 5% sucrose/MES. Samples were centrifuged at 200000g for 20 hours at 4°C. Tubes were removed to ice, and 12 x 1ml fractions were collected from the top. HRP-CTB staining of dot blots of each fraction revealed that fraction 4 and 5 contained lipid rafts (not shown). Fraction 12 was also retained to represent the Triton-soluble fraction. Lipid rafts were collected by centrifugation and solubilized in 1x SDS gel loading buffer (50mM Tris HCl pH6.8, 2% SDS, 10% Glycerol, 0.1% bromphenol blue, 5% -mercaptoethanol).

**Cell fractionation**

1x10^7^ Jurkat TAg cells per assay point were transfected with 10µg cDNA expression plasmids encoding wild-type Coro1A or as expression control pEFneo GFP. After incubation for 21 hours cells were stimulated with solid-phase antibodies against CD3 (DYNAL IgG beads) for 20 min at 37°C or left unstimulated. Cell fractionation was performed by subsequent lysis in different MES lysis buffers (as described above; without Triton, soluble fraction; containing 0.5% Triton X-100, particulate fraction; containing 2% SDS, nonsoluble fraction). 1x10^6^ cell equivalents of the soluble (s) fraction and 1x10^7^ cell equivalents of the particulate (pt) and the nonsoluble fraction (ns) was analysed by immunoblotting.

# Reference

1. Hermann-Kleiter N, Gruber T, Lutz-Nicoladoni C, Thuille N, Fresser F, Labi V, Schiefermeier N, Warnecke M, Huber L, Villunger A, et al: **The nuclear orphan receptor NR2F6 suppresses lymphocyte activation and T helper 17-dependent autoimmunity.** *Immunity* 2008, **29:**205-216.
